# Analysis of Photocatalytic Properties of NS-CQDs/g-C_3_N_4_ Composites

**DOI:** 10.3390/mi14122143

**Published:** 2023-11-23

**Authors:** Yu Wang, Hongyue Chen, Zijian Jia, Jingxue Lv, Yuguang Lv, Jiang Wu

**Affiliations:** 1 College of Pharmacy, Jiamusi University, Jiamusi 154007, China; 18804686070@163.com (Y.W.); chenhongyue2023@163.com (H.C.); lv15146345768@163.com (J.L.); 2 College of Materials Science and Engineering, Jiamusi University, Jiamusi 154007, China; 3 School of Stomatology, Jiamusi University, Jiamusi 154007, China; feichi33479chao@163.com

**Keywords:** CQDs/g-C_3_N_4_, rhodamine B, photocatalytic activity

## Abstract

N- and S-doped CQDs were prepared using L-cysteine as a precursor. Different NS-CQDs/g-C_3_N_4_ composite photocatalysts were formed by modifying graphite-phase carbon nitride with different contents of NS-CQDs using a hydrothermal method. The morphology, constituent elements and functional groups of the composite photocatalysts were analyzed by SEM, EDS, TEM, Mapping, XRD and FT-IR as a proof of its successful preparation. Meanwhile, it was characterized by PL, UV-Vis DRS and electrochemical impedance, which proved that the CQDs could be used as an electronic memory in the composite system to accelerate the electron transfer induced by the photo-excitation of g-C_3_N_4_ and effectively inhibit the recombination of e^−^-h^+^ improvement of the photocatalytic activity of g-C_3_N_4_. The stability of the composite photocatalysts under different conditions and the photodegradation activity of Rh B under visible light were investigated. It was found that the photocatalytic degradation efficiency of rhodamine B by NS-CQDS-modified g-C_3_N_4_ was significantly higher than that of pure g-C_3_N_4_, which could reach 90.82%, and its degradation rate was 3.5 times higher than that of pure g-C_3_N_4_. It was demonstrated by free radical trapping experiments that ·OH and ·O_2_^−^ were the main active species in the photocatalytic degradation process, in which ·O_2_^−^ played a guiding role.

## 1. Introduction

With rapid industrialization and population growth, various pollutants from industrial production are inevitably released into natural water bodies, especially organic pollutants that are difficult to treat (e.g., dyes, pesticides and endocrine-disrupting compounds, pharmaceuticals, and personal care products), and organic dyes discharged into the local environment of various production sites without adequate treatment are one of the important sources of pollution of water sources [1]. There are many types of organic dyes, and they are usually categorized into cationic dyes and anionic dyes according to the ionic state of the dyes when they are dissolved in water. Organic dyes dissolved in water in a cationic state are called cationic dyes, which are also called basic dyes or salt-based dyes because most of their color-generating groups are organic bases. Anionic dyes, also known as acid dyes, can be applied to acidic, weakly acidic or neutral environments. Cationic dyes include Rh B, methyl violet (MV), peacock green and methylene blue (MB) [2,3]. Rh B, as one of the most typical industrial dyes, is also known as a water tracer fluorescent agent, which is widely used in the paper industry, printing and dyeing, textile printing and dyeing, etc. It may cause eye, skin and respiratory problems. It may cause eye, skin, and respiratory and gastrointestinal tract irritation. In 1978, Rh B was declared as a potentially carcinogenic dye by the International Agency for Research on Cancer (IARC). According to the study, the contamination of drinking water with Rh B poses a threat to human health ecosystems, even at a concentration of 1.0 mg/L [4,5]. Dye effluents containing higher concentrations of organic contaminants may contaminate and damage various aquatic systems and may cause carcinogenic and mutagenic changes in organisms. Therefore, in order to prevent further significant environmental problems and harmful risks, large quantities of dyes in wastewater must be treated accordingly before they can be discharged into the environment [6,7,8]. Therefore, there is an urgent need to provide an effective method of wastewater control and treatment.

At present, with the research and development of wastewater treatment technology, various methods and technologies for the treatment of dyes in wastewater are becoming more and more mature, such as adsorption, precipitation, ion exchange, membrane separation, etc. These methods have a certain effect on the removal of heavy metals and dyes in wastewater. However, these methods also have certain shortcomings, which make them have certain limitations in practical application. For example, the use of adsorption and precipitation methods to remove pollutants in wastewater is only to enrich and transfer the target pollutants, which may produce new sources of secondary pollution; in addition, for the ion exchange method and membrane separation method, the cost is high and the efficiency is low, which is not applicable to the large amount of wastewater treatment in industry, and it is difficult to carry out the industrialization application. Compared with the traditional methods, the photocatalytic method, which uses sunlight to excite semiconductors for reaction, has the advantages of easy operation, good adaptability to pollutants and low cost in removing pollutants [9,10,11]. Therefore, the degradation of pollutants using photocatalysts under light conditions is considered to be an effective and most promising method for solving environmental pollution problems. Photocatalytic materials mainly include two categories: molecular catalytic materials and semiconductor materials. Due to their stable structure, simple fabrication and low price, semiconductor-based photocatalysts are more widely explored than molecular catalysts and are gradually becoming the material of choice for photocatalytic treatment of wastewater. Photocatalysts can produce photogenerated electrons and holes under the irradiation of solar energy. These photogenerated electrons and holes react with oxygen, water and hydroxyl groups to produce reactive oxygen species with strong oxidizing ability, such as ·OH and superoxide radical anion (·O_2_^−^). These are the main particles that play a role in the degradation of organic pollutants [12,13,14].

Carbon quantum dots (CQDs), as a newly discovered quasi-zero venomite material, have attracted extensive attention due to their wide absorption band, high light absorption coefficient and chemical stability. In recent years, CQDs’ unique upconversion luminescence properties have been shown to be excited by high wavelength, low frequency light, and then emit high frequency low wavelength light. In addition, CQDs can also act as an electron acceptor to partially reduce the recombination rate of photogenerated electron hole pairs, thus improving the photocatalytic degradation activity of semiconductors [15,16,17]. In recent years, in addition to the combination of CQDs with other nanomaterials, some nonmetallic elements (such as N, P, S, Cl, etc.) have been doped to improve the adsorption and photocatalytic performance. For example, N-doped carbon quantum dots exhibit anomalous photoluminescence outside the visible spectral range and are capable of altering the local electronic structure to increase the capacitance of the CQDs and improve the charge-off domain to enhance the photocatalytic activity of the CQDs. The doping of the S element alters the surface groups and conjugation system of the CQDs to improve the optical properties of the CQDs [18,19,20,21].

Graphite carbon nitride (g-C_3_N_4_), because it is a kind of stable structure of graphite with excellent optical performance and the appropriate band gap structure, has great prospects in the field of photocatalysis; however, because of the pure lump of carbon nitride materials being visible, light utilization efficiency is low and the light of the high raw carrier recombination rate and low specific surface area are restricted by some disadvantages, such as further application. Therefore, how to optimize the structure of g-C3N4 and improve its photocatalytic activity is the current research hotspot [22,23]. By analyzing the photocatalytic mechanism in some typical applications, it can be seen that the factors that have an impact on the photocatalytic performance are mainly as follows: the number of active sites, the absorption rate of the light source, the mobility efficiency of the photogenerated carriers and the complexation rate. Therefore, in order to achieve the goal of improving the photocatalytic activity, scientists began to modify the composition, structure and properties of g-C3N4 materials and optimized their structures using various means [24,25,26], such as morphology modification, formation of conjugated polymers, elemental doping, coupling with other semiconductors, quantum dot modification, coupling carbon materials, introduction of structural defects and co-polymerization [27].

Based on the above characteristics, NS-doped carbon quantum dots (NS-CQDs) and g-C_3_N_4_ composites were prepared, and their RhB degradation and adsorption properties were investigated and the degradation mechanism was probed. This provides scientific theoretical value for the practical application of this composite photocatalyst.

## 2. Materials and Methods

### 2.1. Preparation of Catalyst

The preparation of NS-CQDs: 0.5 g L-cysteine was accurately weighed and dissolved in 30 mL deionized water and kept stirring until dissolved. Next, 1 mL phenylacetaldehyde was added and stirred for another 20 min. Then, the solution was transferred to a PTFE lined reactor and reacted at 180 °C for 6 h to obtain a dark yellow carbon quantum dot solution. The solution was centrifuged to remove bulk solids, and the particles were removed 2–3 times with a 0.22 μm filter membrane. The solution was freeze-dried in a vacuum for 12 h.

The preparation of g-C_3_N_4_: g-C_3_N_4_ was synthesized by thermal polymerization with urea as a precursor. Then, 10 g urea was weighed in an alumina crucible, placed in a Muffle furnace, heated to 550 °C at a rate of 5 °C/min and kept for 4 h. After cooling, it was removed and ground to obtain solid powder.

Preparation of NS-CQDs/g-C_3_N_4_ catalyst: NS-CQDs/g-C_3_N_4_ catalyst was prepared by a hydrothermal method. A certain amount of NS-CQDS aqueous solution was added to 30 mL anhydrous ethanol, in which 0.2 g g-C_3_N_4_ was dispersed in anhydrous ethanol. The mixture was fully mixed by stirring ultrasound for 1 h, and the mixture was transferred to a PTFE lined reactor for reaction at 120 °C for 2 h. The samples were collected by centrifugation and washed with anhydrous ethanol and distilled water 3 times, respectively. The obtained solid samples were placed in an oven and dried at 60 °C for 12 h. The resulting samples were NS-CQDs/g-C_3_N_4_ nanocomposites. The amount of NS-CQDs solution added was then varied as described in the above steps so that the doping rates of 3%, 5% and 7% were recorded as 3NSC-g, 5NSC-g and 7NSC-g, respectively.

### 2.2. Visible Light Catalytic Degradation of Rhodamine B Catalyst Performance Evaluation

Under dark conditions, 20 mg of composite photocatalysts was accurately weighed and dispersed in 50 mL of Rh B (10 mg/L) solution. The dark reaction was carried out with stirring for 60 min to reach adsorption desorption equilibrium. The extracted 5 mL of solution was centrifuged and the upper layer was extracted and placed in a UV-Vis absorption spectrometer for sample determination. The photocatalytic experiments were then carried out on a 250 W photocatalytic degradation unit (BBZM-I) for 180 min. During this period, 5 mL of solution was absorbed by pipette every 60 min for centrifugation and the supernatant was extracted. UV-Vis absorption spectra were tested in the wavelength range of 400–600 nm to obtain the absorption spectra of Rh B at each time point. The instrument model of the UV-Visible Spectrophotometer is UV-2550.

### 2.3. Catalyst Performance Evaluation

Using visible light irradiation as the simulation light source and RhB as the organic pollutant, catalytic degradation was used to evaluate the superior performance. RhB solution with a certain concentration was prepared and stored in the refrigerator. Within the range, the solution was placed in a colorimetric dish with deionized water as the reference solution. RhB solution was determined and UV-Vis spectrum was obtained. RhB solution under UV-Vis (λ_max_ = 553 nm).

η/% = (ρ_0_ − ρ_t_)/ρ_0_ × 100% = (A_0_ − A_t_)/A_0_ × 100%

where η is the degradation rate of pollutants, %; ρ_0_ is the initial mass concentration of pollutants, mg/L; ρ_t_ is the mass concentration of pollutants at the moment of dark adsorption or light exposure t, mg/L; A_0_ is the initial absorbance of pollutants; and A_t_ is the absorbance of pollutants at the moment of dark adsorption or light exposure t.

### 2.4. Optical Performance Testing

The electrochemical impedance (EIS) and photocurrent of the catalyst were tested using a Shanghai CH1660E electrochemical workstation. In a standard three-electrode system, a conductive glass ITO loaded with catalyst was used as the working electrode, a platinum wire electrode as the counter electrode, an Ag/AgCl electrode as the reference electrode and an aqueous Na_2_SO_4_ solution of 0.05 mol/L as the electrolyte.

### 2.5. Free Radical Capture Experiments

To investigate the effect of different active species on RhB degradation, different trapping agents were added to the initial RhB solution (10 mg/L, 50 mL) before dark adsorption, followed by the addition of catalyst for dark adsorption, all at 0.1 mmol of trapping agent, and the rest of the steps were the same as in Section 2.2.

## 3. Results and Discussion

### 3.1. XRD and FT-IR Analysis

**Figure 1:**
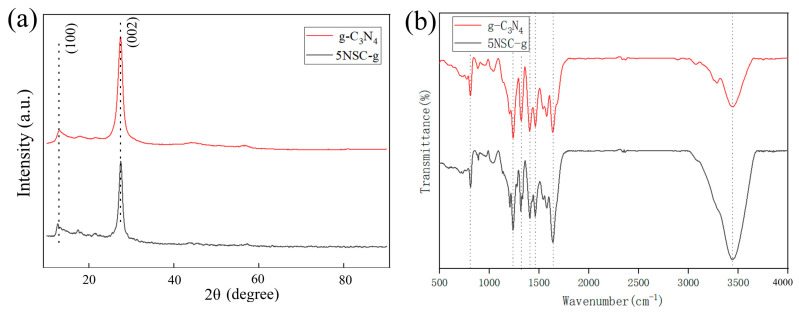
XRD diagram (**a**) and FT−IR spectrum of the catalyst (**b**).

The photocatalysts were characterized and analyzed by XRD and FI-IR using Rigaku Dmax2000 (Tokyo, Japan) and Nicolet iS10 (Waltham, MA, USA). The results of XRD analysis of the g-C_3_N_4_ and NS-CQDs/g-C_3_N_4_ samples are shown in Figure 1a. The crystal structure of g-C_3_N_4_ is well preserved in the g-C_3_N_4_ composites. The g-C_3_N_4_ monomer shows two distinct diffraction peaks at 2θ = 27.2° and 12.7°, which are consistent with the (002) and (100) crystal planes in the standard card (JCPDS No. 871526) [28], respectively. With the increase in NS-CQDs, the position of the (100) diffraction peak was slightly blueshifted toward a lower angular value and the sharpness of the (002) diffraction peak was obviously weakened, which indicated that the NS-CQDs were successfully coupled with g-C_3_N_4_. In addition, the NS-CQDs diffraction peaks were not obvious in the XRD spectra of the NS-CQDs/g-C_3_N_4_ catalysts, which could be attributed to the low crystallinity of NS-CQDs and their high dispersion on the g-C_3_N_4_ surface.

Figure 1b shows a strong absorption peak at 3447.0 cm^−1^, which is typical of hydroxyl and amino peaks, which further indicates a good dispersion in water. It can also be seen that pure g-C_3_N_4_ shows FT-IR absorption peaks at 809.0 cm^−1^, 1242.0 cm^−1^, 1406.0 cm^−1^, 1463.0 cm^−1^, and 1639.0 cm^−1^, which are due to the vibration of the triazine ring of g-C_3_N_4_. This is in agreement with the present presentation in the literature. The peak at 1406.0 cm^−1^ is due to the stretching vibrations of the s-triazine ring unit. This indicates that the g-C_3_N_4_ sintered with urea as the precursor is consistent with that reported in other literature so far, which further indicates that g-C_3_N_4_ has been successfully prepared in this experiment [29]. The FT-IR spectra of g-C_3_N_4_ and 5NSC-g composites are not much different from each other. Several absorption peaks in the range of 1120–1710 cm^−1^ originate from the C-N stretching vibration in the aryl ring, which proves that there are N-(C)_3_ and C-NH-C units in the composites, which form the C-N-C bonding. The absorption peak at 810 cm^−1^ is the bending vibration peak of the triazine ring of g-C_3_N_4_, which indicates that the graphite-phase carbon-nitride-based materials have been successfully synthesized and the chemical structures of g-C_3_N_4_ and 5NSC-g are similar. It can be found that the vibrational broadband of the stretching vibration of -NH belonging to g-C_3_N_4_ exists in the range of 3000–3500 cm^−1^ for the 5NSC-g materials, and all of them have similar absorption peaks [15,30,31]. Meanwhile, the vibrational bending degree of the carbon skeleton was enhanced with the increase in NS-CQDs, and the characteristic vibration of g-C_3_N_4_ was also enhanced, which indicated that the NS-CQDs/g-C_3_N_4_ composites have been successfully prepared.

### 3.2. SEM and EDS Analysis

**Figure 2:**
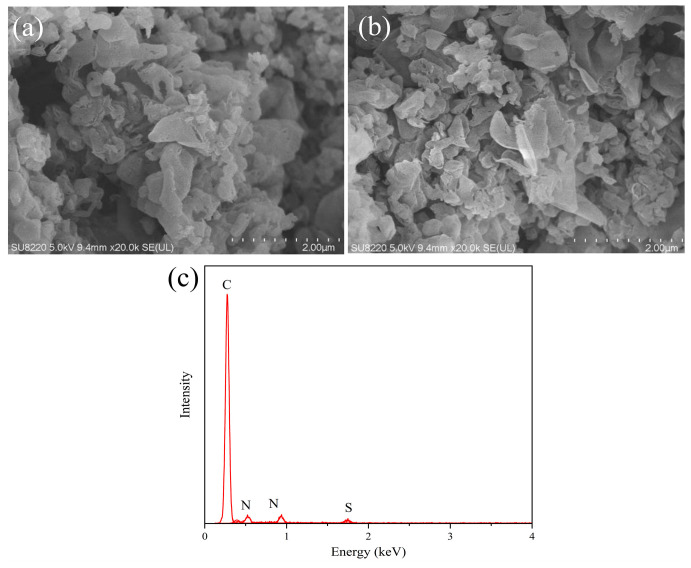
(**a**) SEM diagram of g-C_3_N_4_ and (**b**)NS-CQDs/g-C_3_N_4_; (**c**)EDS diagram of NS-CQDs/g-C_3_N_4_.

SEM tests were performed on the photocatalysts using a Dutch Phenom ProX. Figure 2 shows the SEM images of the prepared samples. In Figure 2a, many lamellar g-C_3_N_4_ can be observed, and the curling phenomenon occurs due to the thin lamellae. Meanwhile, comparing the effect of composite NS-CQDs on the morphology of the material, in Figure 2b, it can be seen that the composite material still maintains a thin lamellar structure after doping with NS-CQDs, and it has a larger specific surface area, which is able to adsorb more substrates. Figure 2c shows the EDS diagram of the composite, which indicates that the composite contains three elements, C, N, and S, indicating the presence of NS-CQDs and g-C_3_N_4_ in the prepared composite catalyst.

### 3.3. TEM and Mapping

**Figure 3:**
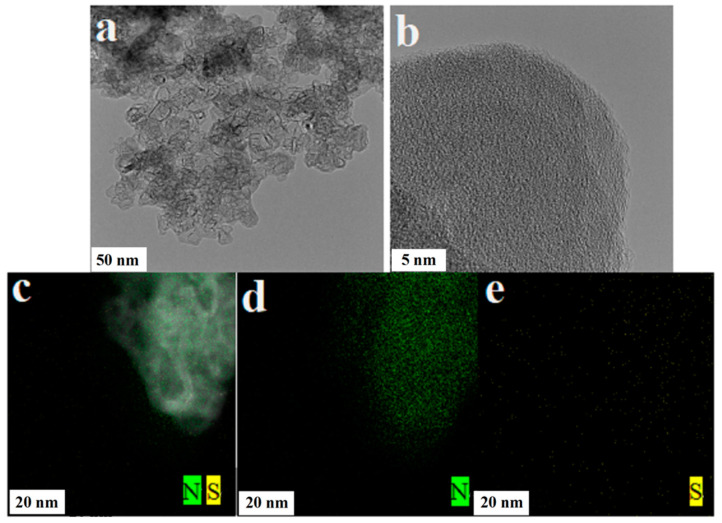
TEM images (**a**,**b**), elemental mapping images (**c**), N-element (**d**), and S-element (**e**) of NS-CQDs/g-C_3_N_4_.

The photocatalysts were subjected to TEM testing using an FEI Talos F200X and Mapping tests were performed with the included energy spectrometer. Figure 3 shows the TEM images and mapping surface sweeps of NS-CQDs/g-C_3_N_4_. From Figure 3a,b, it can be seen that the prepared NS-CQDs/g-C_3_N_4_s present a thin sheet structure and have transparent properties, indicating that the composite material with ultra-thin lamellae of g-C_3_N_4_ as the main body has been successfully prepared and, due to the weak relative lining between the NS-CQDs and the g-C_3_N_4_ lamellae, the presence of the NS-CQDs could not be observed. However, the mapping surface of NS-CQDs/g-C_3_N_4_ can be swept in Figure 3c–e to reveal that the NS-CQDs/g-C_3_N_4_ composite nanomaterials contain N and S and are uniformly distributed, so it can be demonstrated that the NS-CQDs are successfully loaded on the g-C_3_N_4_ surface.

### 3.4. UV-Vis DRS Analysis

**Figure 4:**
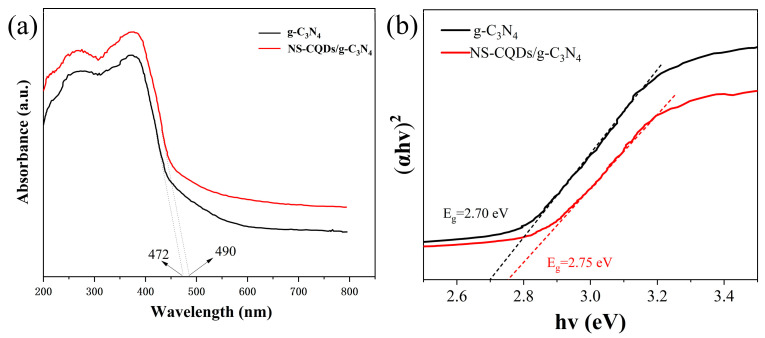
UV-Vis DRS spectra (**a**) and plots of (αhv)^2^ versus energy (hv) (**b**) of g-C_3_N_4_ and 5NSC-g.

UV-Vis DRS tests were performed on the photocatalysts using a PE lambda 750. The UV-Vis DRS can indicate the light absorbance of the prepared samples. Figure 4a shows the DRS plots of g-C_3_N_4_ and 5NSC-g, from which it can be seen that the absorption threshold of g-C_3_N_4_ occurs at 472 nm and that of 5NSC-g occurs at 490 nm and the comparison can be seen that the complexation of NS-CQDs makes the visible light absorption of g-C_3_N_4_ improved. The respective forbidden bandwidth (Eg) values were calculated according to the Kubelka–Munk function, as shown in Figure 4b, and the Eg values of g-C_3_N_4_ and 5NSC-g were 2.70 eV and 2.75 eV, respectively. Thus, the photocatalytic performance of g-C_3_N_4_ was improved.

### 3.5. PL Analysis

**Figure 5:**
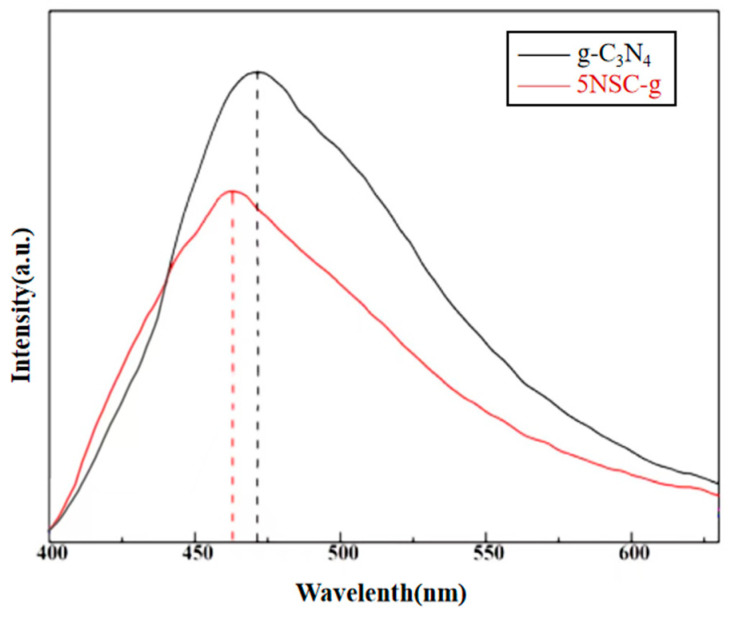
PL spectrum of the catalyst.

Photoluminescence (PL) spectroscopy reveals the generation, migration, separation and compounding behavior of photogenerated charges in semiconductor photocatalyst materials during the photocatalytic process. Usually, the weaker the fluorescence intensity, the higher the efficiency of photogenerated electron–hole pair separation. We tested the performance of the composite photocatalysts using a fluorescence spectrophotometer (F2500). Figure 5 shows the PL plots of g-C_3_N_4_ and NS-CQDs/g-C_3_N_4_, respectively. As can be seen from the figure, the PL intensity of NS-CQDs/g-C_3_N_4_ composite photocatalyst is significantly lower than that of g-C_3_N_4_. This is mainly caused by the excellent conductivity of NS-CQDs, which accelerates the migration rate of photogenerated charges. This indicates that the introduction of NS-CQDs can inhibit the complexation of photogenerated electron–hole pairs and further improve the photocatalytic activity.

### 3.6. Optical Performance Analysis

**Figure 6:**
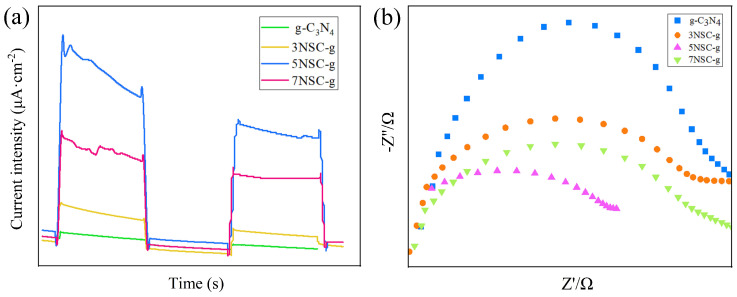
Photoelectric response (**a**) and electrochemical impedance spectrum (**b**) of the sample under visible light irradiation.

To further investigate the photogenerated charge migration and separation efficiency, the transient photocurrent response (I-T) and electrochemical impedance spectra (EIS) of g-C_3_N_4_ and NS-CQDs/g-C_3_N_4_ were tested under visible light (λ > 420 nm) irradiation (Figure 6). Figure 6a shows the transient photocurrent response profiles for the switching lamp cycle. It can be seen that the photocurrent density is maintained at a relatively stable value under the on-lamp condition; when the lamp is turned off instantaneously, the photocurrent density drops rapidly. 5NSC-g has the strongest photocurrent density. In general, the higher the photocurrent density, the higher the separation efficiency of photogenerated electron–hole pairs. 5NSC-g has the strongest photocurrent density, indicating that it has a more efficient photogenerated electron–hole pair separation efficiency and a longer electron lifetime than g-C_3_N_4_ and, thus, has better photocatalytic activity.

The relative size of the electrode arc radius was g-C_3_N_4_ > NS-CQDs/g-C_3_N_4_. Since the smaller the arc radius of the EIS map, the lower the charge transfer resistance and the faster the photogenerated carrier transport capacity, the electrochemical impedance spectroscopy results from g-C_3_N_4_ and NS-CQDs/g-C_3_N_4_ (Figure 6b) further demonstrate that, in NS-CQDs/g-C_3_N_4_, the electrotransfer efficiency is higher than that of g-C_3_N_4_, which is consistent with the transient photocurrent response results.

### 3.7. Effect of Different NS-CQDs Doping on RhB Degradation

**Figure 7:**
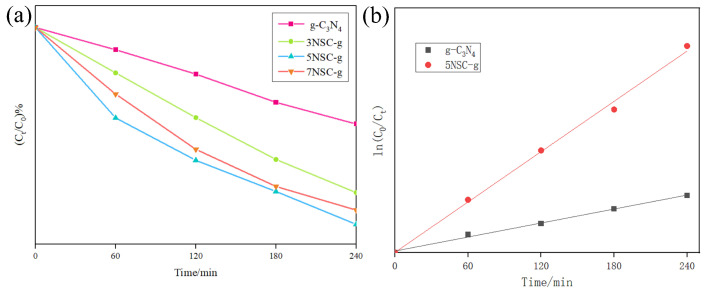
Effect of different NS-CQDs doping on RhB degradation (**a**); kinetic analysis of photocatalytic reactions (**b**).

Figure 7a shows that, when only g-C_3_N_4_ is present in the reaction, only 44.54% of RhB is degraded after 4 h of reaction, indicating that g-C_3_N_4_ has a weak degradation effect on RhB under the condition of visible light. Compared with g-C_3_N_4_, all NS-CQDs/g-C_3_N_4_ samples showed higher photodegradation activity of RhB. With the increase in loaded NS-CQDs, the degradation ability of RhB by NS-CQDs/g-C_3_N_4_ increased first and then decreased. When the addition amount of NS-CQDs was 3 mL, the degradation rate of RhB could reach 90.82% after 4 h of reaction. However, when the amount of NS-CQDs was increased, the photodegradation ability of the catalyst was weakened because too much NS-CQDs covered the surface of g-C_3_N_4_ and occupied the active site of g-C_3_N_4_, which reduced the performance of the catalyst. At the same time, excessive NS-CQDs will also compete with g-C_3_N_4_ for photons, resulting in an internal filtering effect on light, and thus reduce the generation of photogenerated electron–hole pairs.

The first-order kinetic equation of photocatalytic degradation of RhB by g-C_3_N_4_ and 3NSC-g was fitted, and the first-order kinetic equation was as follows. The results are shown in Figure 7b.

ln(C_0_/C_t_) = kt (1)

where t—reaction time, (min); K—first-order kinetic constant, (min^−1^); C_0_—initial concentration of RhB, (mg/L); and C_t_—RhB concentration at reaction time t, (mg/L).

According to Formula (1), the first-order kinetic parameters calculated by fitting are shown in Table 1. According to the R^2^ value (>0.99) in Figure 7b and Table 1, the first-order kinetic equation can fit the kinetic process of photocatalyst degradation of RhB. Among them, the degradation rate of RhB of 5NSC-g composite photocatalyst is 3.5 times that of pure g-C_3_N_4_.

### 3.8. Free Radical Capture Experiments

**Figure 8:**
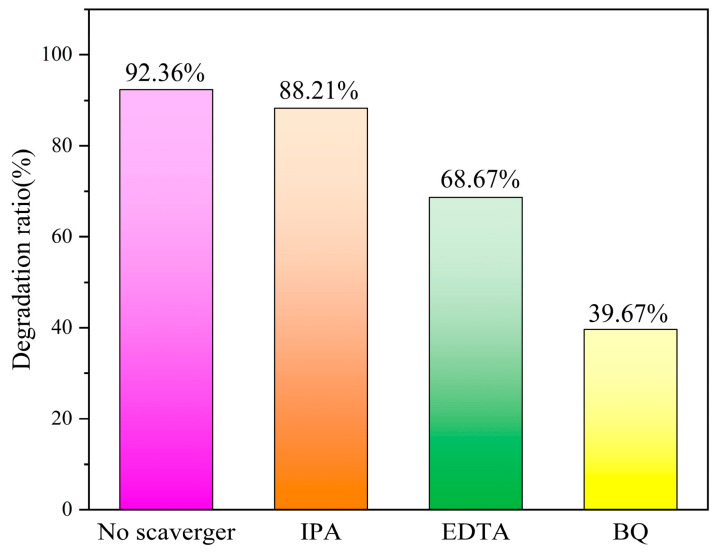
Effect of different radical trapping agents on catalytic degradation agents.

The photocatalytic degradation process generates different kinds of reactive radicals and, in order to identify the radicals that play a major role in the photocatalytic degradation of the composites, free radical capture experiments were performed, as shown in Figure 8. Based on the photocatalytic degradation experiments, isopropanol (IPA), sodium oxalate and benzoquinone (BQ) were added to the reaction solution to capture the hydroxyl radicals ·OH, hole h^+^ and superoxide radicals ·O_2_^−^ generated in the reaction system, respectively. The results showed that the degradation rate of RhB hardly decreased after the addition of IPA, indicating that the generation of ·OH was very low. The photocatalytic efficiencies of the NS-CQDs/g-C_3_N_4_ nanocomposite catalysts were both significantly reduced after the introduction of EDTA and BQ, so the captured h^+^ and superoxide radical ·O_2_^−^ were more than the hydroxyl radical ·OH. From this, it can be inferred that the photodegradation of RhB over NS-CQDs/g-C_3_N_4_ nanocomposite catalysts is mainly caused by h^+^ and ·O_2_^−^, and the generated ·O_2_^−^ has a greater effect on the photocatalytic degradation than h^+^.

### 3.9. Analysis of Photocatalytic Reaction Mechanism

**Figure 9:**
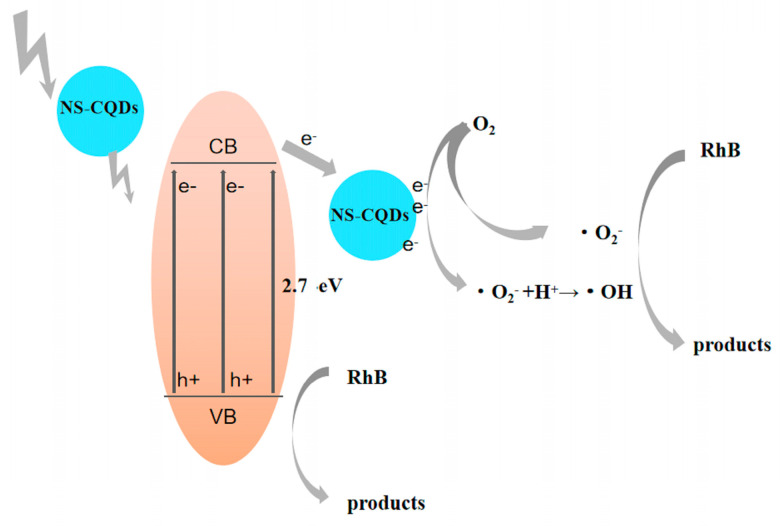
The photocatalytic degradation mechanism of RhB.

Figure 9 shows the reaction principle of NS-CQDs/g-C_3_N_4_ photocatalytic system. The reaction principle of NS-CQDs/g-C_3_N_4_ photocatalytic system is shown in Figure 9. The modification of g-C_3_N_4_ by nitrogen-doped carbon quantum dots can effectively improve the photocatalytic activity of g-C_3_N_4_, which can be attributed to the efficient electron transfer property of NS-CQDs, which promotes the photogenerated carrier separation efficiency of g-C_3_N_4_ and facilitates the transfer of photogenerated electrons from the conduction band to the surface of g-C_3_N_4_ to adsorb ·O_2_^−^, forming superoxide anion radical ·O_2_^−^ active species, thus promoting the photocatalytic efficiency improvement. The results of photocatalytic performance experiments showed that too many NS-CQDs would form complex centers and weaken the photocatalytic activity of NS-CQDs/g-C_3_N_4_ photocatalysts.

## 4. Conclusions

In this study, NS-CQDs were compounded with g-C_3_N_4_ as substrate and NS-CQDs/g-C_3_N_4_ photocatalytic degradation materials were prepared by hydrothermal synthesis. SEM, EDS, TEM, and Mapping characterizations were performed to prove the successful preparation of the material. From the UV-Vis DRS test, it can be concluded that the modification of NS-CQDs can effectively broaden the visible light response of g-C_3_N_4_ and shorten the forbidden bandwidth. From the characterization of photoluminescence spectra, it can be found that the modification of nitrogen–sulfur co-doped carbon quantum dots can effectively transfer photogenerated electrons and well separate photogenerated electron–hole pairs. The composite photocatalysts were applied to RhB and the degradation rate and degradation mechanism were explored. It was experimentally concluded that the degradation rate of RhB by NS-CQDs/g-C_3_N_4_ composite photocatalyst could reach 90.82%, and its degradation rate was 3.5 times higher than that of g-C_3_N_4_ alone. From the free radical trapping experiments, it was concluded that ·O_2_^−^ and h^+^ played important roles in the photocatalytic degradation of RhB by NS-CQDs/g-C_3_N_4_, with ·O_2_^−^ as the dominant.

## Figures and Tables

**Table 1:** Results of photocatalytic reaction kinetic analysis.

Sample Type	Regression Equation	k	R^2^
g-C_3_N_4_	y = 0.0029x + 0.0186	0.0029	0.9955
5NSC-g	y = 0.0103x + 0.0023	0.0103	0.9962

## Data Availability

The data is unavailable due to privacy or ethical restrictions.
